# Using MapMyFitness to Place Physical Activity into Neighborhood Context

**DOI:** 10.3389/fpubh.2014.00019

**Published:** 2014-03-11

**Authors:** Jana A. Hirsch, Peter James, Jamaica R. M. Robinson, Kyler M. Eastman, Kevin D. Conley, Kelly R. Evenson, Francine Laden

**Affiliations:** ^1^Department of Epidemiology, Center for Social Epidemiology and Population Health, University of Michigan School of Public Health, Ann Arbor, MI, USA; ^2^Department of Epidemiology, Harvard School of Public Health, Boston, MA, USA; ^3^Department of Environmental Health, Harvard School of Public Health, Boston, MA, USA; ^4^MapMyFitness, Inc., Austin, TX, USA; ^5^Department of Electrical Engineering, Stanford University, Stanford, CA, USA; ^6^Department of Epidemiology, University of North Carolina Gillings School of Global Public Health, Chapel Hill, NC, USA; ^7^Department of Medicine, Channing Division of Network Medicine, Brigham and Women’s Hospital, Harvard Medical School, Boston, MA, USA

**Keywords:** physical activity, GPS, quantified self, big data, recreation, parks, MapMyFitness, MapMyRun

## Abstract

It is difficult to obtain detailed information on the context of physical activity at large geographic scales, such as the entire United States, as well as over long periods of time, such as over years. MapMyFitness is a suite of interactive tools for individuals to track their workouts online or using global positioning system in their phones or other wireless trackers. This method article discusses the use of physical activity data tracked using MapMyFitness to examine patterns over space and time. An overview of MapMyFitness, including data tracked, user information, and geographic scope, is explored. We illustrate the utility of MapMyFitness data using tracked physical activity by users in Winston-Salem, NC, USA between 2006 and 2013. Types of physical activities tracked are described, as well as the percent of activities occurring in parks. Strengths of MapMyFitness data include objective data collection, low participant burden, extensive geographic scale, and longitudinal series. Limitations include generalizability, behavioral change as the result of technology use, and potential ethical considerations. MapMyFitness is a powerful tool to investigate patterns of physical activity across large geographic and temporal scales.

## Introduction

Physical activity plays a role in the etiology of numerous chronic diseases, including cancer and cardiovascular disease (1, 2). Tracking where, when, and by whom physical activities occur could clarify the ways that public health can encourage more activity and lower chronic disease risk. However, to date, lack of fine-grain geographic data have limited research into national spatial patterns of physical activity. Fitness apps, seven of which reached at least 16 million downloads apiece as of August 2013, could act as tools to supply this type of data to health research (3).

Increasingly, individuals in the United States are turning to technology in order to monitor and manage their health. As of 2013, cell phone ownership among adults exceeds 90% (4, 5), and according to different estimates, over 60% use smartphones (5–7). Mobile phones have entered into numerous research contexts, particularly because of the rich dynamic spatial information they can provide (8). Self-tracking by individuals, particularly of health and fitness information, has become increasingly common. Nineteen percent of all mobile internet users have downloaded a fitness or health app and 9–11% have integrated that app into their daily lives (9). By monitoring their routes and workouts through an app, consumers passively contribute their logs to a non-specific, multi-regional data pool (10, 11). The use of these health apps, many of which include a built-in global positioning system (GPS), enables the analysis of individual and group fitness trends across broad large spatial scales (12–14).

In the past, studies exploring spatial patterns in physical activity using personal sensors have often been designed from a researcher-driven perspective (15, 16). Investigators assigned participants a personal sensor and asked them to self-report behaviors over time (15, 17, 18). Due to the effort required to collect data, the specialized nature of the datasets, and the limited geographic areas in which it was feasible to conduct the research, these studies have resulted in limited generalizability (19).

MapMyFitness is a suite of interactive tools for individuals to track their workouts online or using GPS in their phones or other wireless trackers. Our intent is to present an illustration of how data tracked using MapMyFitness can be applied to the investigation of physical activity patterns over space and time. In doing so, we will emphasize the potential benefits associated with the use of this technology as a powerful tool in scientific research. Finally, we describe the conceivable limitations and ethical concerns involved in using these data to deepen our understanding of the interplay between context and physical activity (20–22).

## MapMyFitness Description

MapMyFitness^1^ provides interactive tools for individuals to track their workouts. MapMyFitness was started in July 2005, as “MapMyRun.com.” In December 2006, MapMyFitness was created. By April 2007, the MapMyRide, MapMyFitness, MapMyWalk, and MapMyHike websites were all made available to the public and by September 2008, MapMyRide and MapMyRun were among the first 200 iPhone^®^ apps in the App Store. As of October 2013, MapMyFitness had a community of over 20 million registered users.

MapMyFitness is an open platform that integrates with more than 400 fitness tracking devices, sensors, and wearable trackers. Users can track workouts and plot the route of walks, runs, and bicycle rides, among other activities. Route data are collected using GPS within the mobile app, by manual mapping through their website, and through linked devices, such as Garmin GPS monitors. Approximately 97% of all routes are tracked via GPS and the mobile app, rather than recorded manually by users online. Users can save the route and share it with the MapMyFitness community or with other social media outlets. A route can then be re-used by that user, or another user, for additional workouts. The MapMyFitness basic features are free or users can upgrade to an “MVP” membership to unlock additional benefits such as advanced heart rate analytics, mobile coaching, training plans, route recommendations, and live tracking to social media. While 74% of the routes tracked by October 2013 were within the United States, users recorded routes throughout the world (23). To date, there are over 197900000 workouts logged, covering over 1005900000 miles and more than 163700000 h.

### Technical details

**Figure 1:**
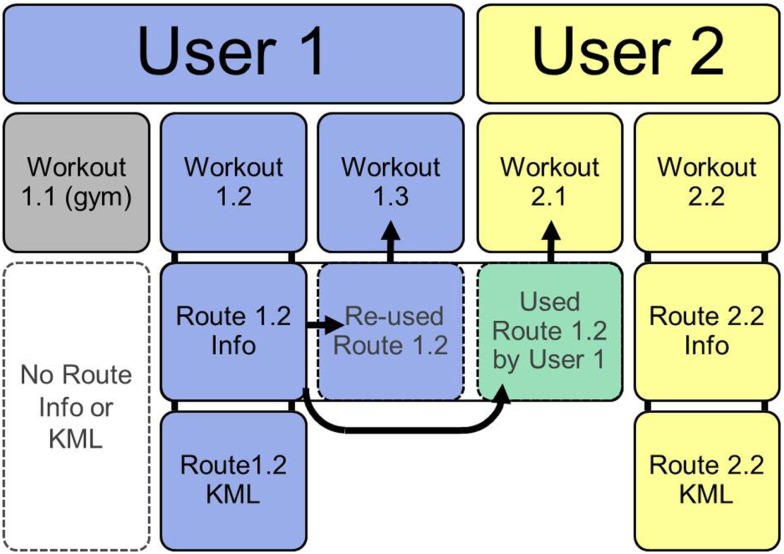
**Structure of MapMyFitness data**. If users log workouts that do not have geographic information (e.g., in a gym) no route information or route KML is created (workout 1.1). Most workouts are logged by tracking a route. This creates a route information file and a route KML of the geographic path (workout 1.2 and 2.2). Once a route is saved, it can be re-used by the same user for a new workout (workout 1.3) or by another user for a new workout (2.1).

Some data [e.g., age group, sex, and body mass index (BMI)] are input and updated by users, while other data (e.g., route path and speed) are recorded and calculated by the MapMyFitness suite. Data from MapMyFitness are stored across three domains: workouts, routes, and users (Figure 1). Workouts represent a specific instance of physical activity. Each workout includes a route identification number, if applicable, and a user identification number. Workouts that are tracked in a gym for strength training or on a treadmill do not have a route. Workouts include information on start dates and times, duration, distance, type, estimated calories, and speed. Estimated calories are calculated from corrected Metabolic Equivalent (METS), which first estimate a resting metabolic rate based on age, gender, height, and weight (24). Then, the type of activity, speed, and duration are factored in to a multiplier of the resting metabolic rate, giving an estimate of calories burned for each activity.

As of October 2013, public data could be downloaded from MapMyFitness using an API (Application Programing Interface) to directly search and download workouts, routes, or users. Alternately, we contacted MapMyFitness directly to acquire a larger dataset for specific locations and years. Data are provided in Comma Separated Value (CSV) format with one row per workout. Routes are available in two formats: a CSV of route information and a Keyhole Markup Language (KML) of the geographic path taken during the route. The route CSV includes a user identification number and route type. Geographic data are represented by a route KML file with the route identification number as the name. The KML stores latitude, longitude, and altitude of each point along a route. For researchers who aim to combine the route geographic information with ArcGIS software (ESRI, Redlands, CA, USA), individual KML files can be imported into ArcGIS. Alternatively, in this paper, we opted to convert points from KML files to DBF using Python (Python Software Foundation. Python Language Reference, version 2.7 available at www.python.org). User information is provided in a CSV format that includes one row for each user with an identification number, sex, age group, and BMI.

## Application to Physical Activity Research (Implementation)

MapMyFitness has numerous applications to investigate physical activity within large-scale geographic and temporal contexts. The widespread adoption of GPS fitness tracking provides a picture of broad geographic physical activity patterns, across the United States and internationally. It therefore allows for substantially larger samples of physical activity behavior and location than have been previously available across time and space. International analyses would allow researchers to understand broad societal influences on physical activity while also identifying common small-scale cues for increasing physical activity.

The fine resolution GPS data provided by MapMyFitness users also allows researchers to link geocoded physical activity information to other geographic features for specific dates and times. These linkages enable researchers to understand where individuals obtain physical activity, as well as to identify specific features that might serve as barriers or enablers for physical activity. This facilitates research exploring not only large-scale physical activity patterns by region, but also the influence of small-scale factors such as neighborhood socioeconomic status, built environment features, or parks and green space.

**Figure 2:**
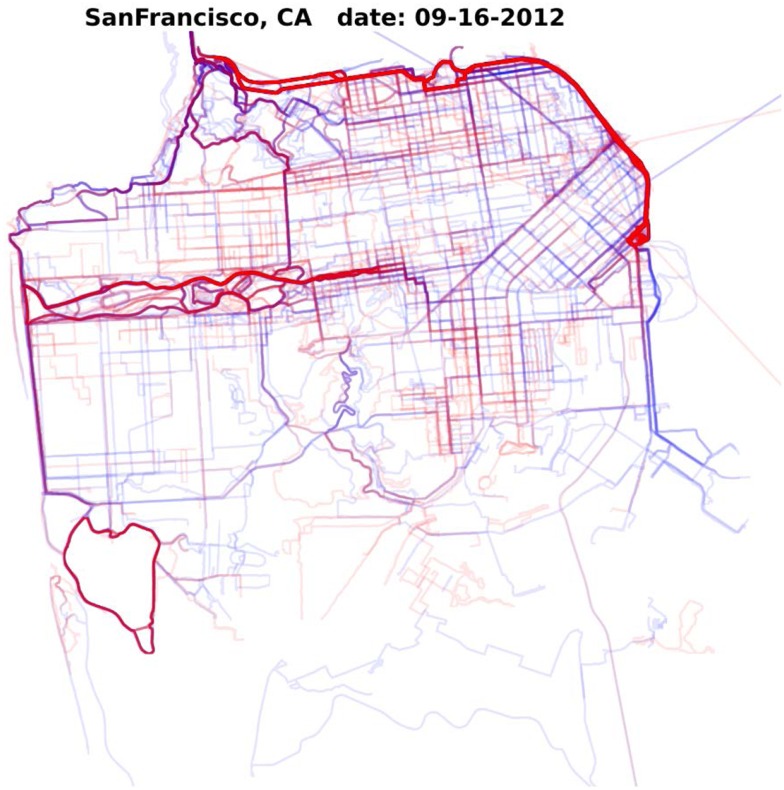
**Density of MapMyFitness routes in San Francisco, CA, USA on September 16, 2012 by sex**. Blue represents routes by male MapMyFitness users, red represents routes by female MapMyFitness users. Thicker lines indicate more routes.

Physical activity patterns can also be examined by different individual-level factors, such as age, sex, and BMI. MapMyFitness can be used to disaggregate the way that individual-level characteristics shape the environment’s influence on physical activity. As illustrated in Figure 2, patterns of physical activity can be examined geographically by sex to identify locations in which each sex is more likely to exercise. Similar analyses could investigate the locations that different age or BMI groups are most likely to traverse. MapMyFitness data could potentially be used as a unique way to augment surveillance data, such as the National Health and Nutrition Examination Survey (NHANES) and other repeated cross-sectional studies, to explore longitudinal fine-grained location data within a national context.

The ability to observe physical activity across large temporal and spatial scales also lends itself to evaluations of policy and environmental interventions to improve physical activity. Researchers could examine patterns of physical activity before and after policy changes at the local, state, national, or even international level. At a local level, field work could identify design changes in the environment and then evaluate their effects based on subsequent changes that occurred in MapMyFitness routes. At a state level, researchers could compare municipalities with and without complete streets or housing policies, as well as trends pre- and post-policy adoption. At a national level, researchers could compare physical activity trends between different regions to estimate the effectiveness of active living programs, such as Center for Disease Control and Prevention’s Community Transformation Grants (25). MapMyFitness data also have the potential to be used in Health Impact Assessments (HIAs) to establish baseline levels of physical activity and to determine which populations will be impacted by changes to policies or the environment. For example, if a HIA was conducted on improvements to an urban park, MapMyFitness data could be used to demonstrate baseline levels of physical activity taking place in the park. Researchers and policy makers could also capitalize on MapMyFitness data during the monitoring and evaluation phase of the HIA to estimate shifts in physical activity taking place in the park after the improvements.

## Example

### Background

Park access has been shown to be an important correlate of physical activity, and the creation of new parks is a suggested intervention to increase physical activity levels in the United States (26, 27). Recent research has begun to use GPS to assess where physical activity occurs (11, 28) and describe patterns of physical activity in parks among participants who are asked to wear both accelerometer and GPS devices (29). Using data from a self-tracker, such as MapMyFitness, allows for an investigation of the links between parks and physical activity over long time periods and with a larger sample.

### Objectives

This example documents MapMyFitness users and characteristics of their physical activity in Winston-Salem, NC, USA from 2006 through 2013. This example then uses MapMyFitness to examine what percent of tracked physical activity occurred in parks and compares characteristics of users and physical activity by park use.

### Methods

**Figure 3:**
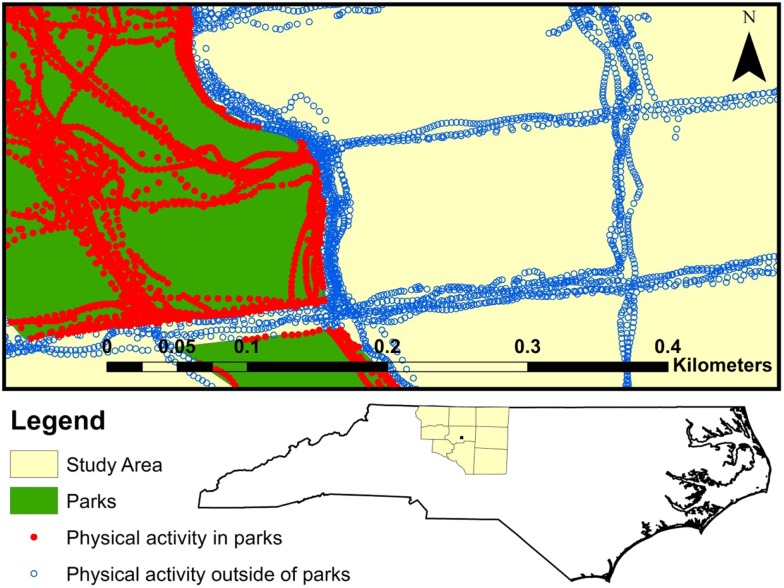
**Winston-Salem study area included in analysis (Davie, Davidson, Forsyth, Guilford, Rockingham, Stokes, Surry, Yadkin, and Randolph counties)**. A sample of MapMyFitness points within the Winston-Salem study area, colored by whether they are in a park.

County boundaries were used to delineate the Winston-Salem study area, which included Davidson, Davie, Guilford, Forsyth, Randolph, Rockingham, Stokes, Surry, and Yadkin County, NC, USA (1837 miles^2^) (Figure 3). Parks were defined as public places set aside for physical activity and enjoyment. Cemeteries, mobile home parks, historic sites, professional stadiums, country clubs, zoos, private parks, private facilities (such as stand-alone baseball or tennis facilities), and stand-alone recreation centers were not included in this definition. Park data were collected as part of the Multi Ethnic Study of Atherosclerosis (MESA). Neighborhood study using two methods. First, we contacted municipal and county GIS, planning, and parks and recreation offices to acquire electronic copies of park files from 2009 to 2012. The parks data were assembled into shape files, which included the name and two-dimensional outline of each park, drawn as a polygon. In a few instances, we drew the park boundary using Google maps when no other outline of the park was available. If only part of the polygon for a confirmed park was in the study area, it was retained. Parks with multiple polygons but the same name were manually merged and assigned as one park. Second, we assembled commercial park shape files from the 2010 ESRI file. The metadata (a summary statement or document containing information on the data set) indicated that parks and forests were identified at the national, state, and local level, including county and regional parks, and referenced Tele Atlas MultiNet North America. All parks were verified similarly to the municipal/county sources, mainly through online searching or phone inquiries and removed if it did not meet our park definition. More details are provided elsewhere and this method of combining commercial and municipal/county data sources provides the most complete and accurate geographic data on parks (30).

Workouts (*n* = 85765), routes (*n* = 74298 in a route information CSV, *n* = 93384 route KML files), and user information (*n* = 4312) for Winston-Salem, NC, USA were obtained from MapMyFitness, Inc. Data included workout information (user, route, workout date, workout type, duration, distance, estimated calories, and speed), route KML files, route information (user, route name, route type, route distance, and city/state), and user information (sex, age group, BMI category, and joining date). Types of activities included in the workout information were run, walk, hike, bicycle ride, swimming, sports/activities, and gym/health club. BMI categories were designated by MapMyFitness as underweight (<18.5), normal weight (18.5–24.9), overweight (25.0–29.9), and obese (≥30.0) (31). Discrepancies between the number of records in each data type arose since data were pulled from the main MapMyFitness database by geographic location (route KML) or by city name (route, user, and workout information). For example, a route KML may have been pulled from the MapMyFitness database because it fell within the geographic boundaries of Winston-Salem; however, if the user did not write “Winston-Salem” as the location of the workout when tracking the route, the route may not appear in the route CSV. Workouts were included in this analysis if they had corresponding user and route information, and if they were entirely contained within the collected study area (*n* = 46248). This restriction resulted in a sample of routes that were geographically within the study area, were coded as being in Winston-Salem for the route and the workout, and were logged by users who indicated they lived in Winston-Salem. Workouts were excluded if speed was ≤0 or >20 mph for walks, runs, hikes, swims, or sports (*n* = 1418) or >50 mph for bicycle rides (*n* = 191) and if distance recorded was more than 1 mile different between the route and workout file (*n* = 386). We further restricted to only adult users ≥18 years of age, excluding 381 workouts and 375 routes performed by 67 youth or adolescent users <18 years of age, leaving a final sample size of 43872 unique workouts on 42003 unique routes by 3094 unique users.

We calculated means and frequencies among workouts’, routes’, and users’ characteristics overall and by time period. We divided the data into an early time period (2006–2009), representing early MapMyFitness adopters, and later time period (2010–2013), coinciding with when the majority of MapMyFitness users joined. Route KML files were mapped in ArcGIS. Each route line was divided into its component points and intersected with park data. This process indicated whether each point was located inside or outside of a park. Figure 3 illustrates a sample of route points within the study area, colored by whether the point is in a park. We calculated percent of points inside a park and compared characteristics of routes that did not enter any parks (0% in parks), were in parks >0% but <50% of the route, were in parks for 50% or more of the route but less than the entire time, and were entirely within parks (100% in parks). Chi-square tests, Analysis of Variance (ANOVA), or Kruskal–Wallis non-parametric tests were used to test for differences across categories as appropriate. All statistical analyses were done in SAS 9.2 (Cary, NC, USA).

### Results

**Figure 4:**
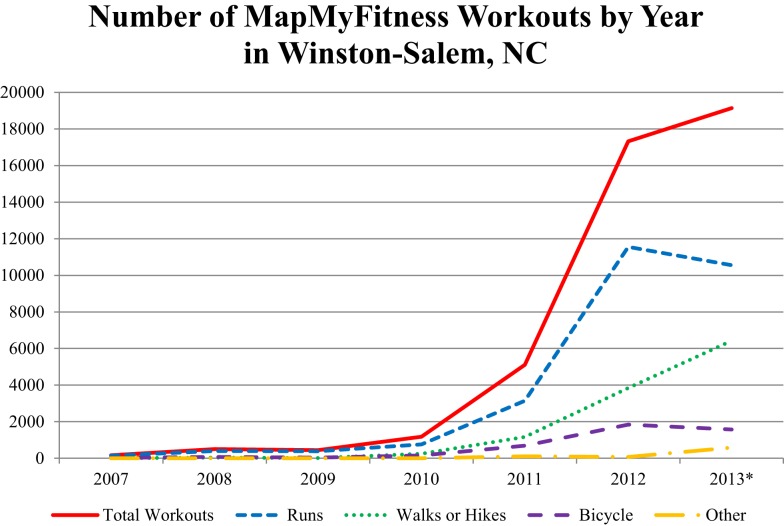
**Time-trends in MapMyFitness workout data for Winston-Salem, NC, USA by workout type**. *Data for 2013 only represents January 1, 2013 through September 24, 2013.

MapMyFitness workouts included in this analysis ranged in time from April 28, 2007 to September 24, 2013. Time-trends in the Winston-Salem MapMyFitness data showed that the number of MapMyFitness workouts increased exponentially starting in 2010 (Figure 4). User joining date ranged from June 15, 2006 to September 23, 2013. A majority of users joined after 2010, with only 7.2% of users joining between June 2006 and December 2009 and 92.8% joining between January 2010 and September 2013.

Of the 43872 unique workouts, 61.4% were runs, 26.7% were walks or hikes, 10.0% were bicycle rides, and 1.8% were other (Table 1). On average, workouts lasted for 46.3 minutes [Standard Deviation (SD) 120.7]. Workouts that were runs, walks, or hikes traveled a mean of 3.3 miles (SD 2.3) at an average speed of 5.1 mph (SD 1.7). Bicycle workouts traveled a mean of 13.9 miles (SD 10.6) at an average speed of 11.8 mph (SD 3.8). Other workouts traveled a mean of 3.7 miles (SD 4.4) at an average speed of 5.3 mph (SD 2.9). Overall, workouts burned a mean of 394.0 estimated calories (SD 536.8). Workouts logged in the earlier time period (between 2007 and 2009) were more likely to be runs or bicycle rides, be longer in terms of both distance and time, and be faster.

**Table 1:** **Characteristics of MapMyFitness workouts, routes, and users within Winston-Salem, NC, USA overall and by time period (June 2006–September 2013)**.

	Overall	Early (2006–2009)^a^	Late (2010–2013)^a^

	**Mean (SD) or percent (*n*)**	**Mean (SD) or percent (*n*)**	**Mean (SD) or percent (*n*)**
**Workouts (*n*)**	43872	1133	42739
Workout type
Run	61.4% (26946)	82.4% (934)	60.9% (26012)
Walk or hike	26.7% (11743)	4.9% (55)	27.3%(11688)
Bicycle	10.0% (4408)	12.7% (144)	10.0% (4264)
Other^b^	1.8% (775)	- - - - - - -^b^	1.8% (775)
Distance (mi)
Run, walk, or hike mean	3.3 (2.3)	5.3 (3.9)	3.3 (2.2)
Bicycle mean	13.9 (10.6)	22.2 (14.6)	13.6 (10.4)
Other mean	3.7 (4.4)	- - - - - - -^b^	3.7 (4.4)
Speed (mph)
Run, walk, or hike mean	5.1 (1.7)	6.4 (1.6)	5.1 (1.7)
Bicycle mean	11.8 (3.8)	15.0 (2.2)	11.7 (3.8)
Other mean	5.3 (2.9)	- - - - - - -^b^	5.3 (2.9)
Time taken (min)	46.3 (120.7)	69.4 (222.6)	45.7 (127.3)
Estimated calories burned (kcal)	394.0 (536.8)	605.1 (687.5)	388.3 (531.0)
**Routes (*n*)**	42003	794	41209
Park information
Not touching any park	71.3% (29943)	78.0% (619)	71.2% (29324)
Entirely in a park	2.9% (1228)	0.3% (2)	3.0% (1226)
Percent of route in a park	11.1 (27.1)	2.9 (11.4)	11.2 (27.3)
**Users (*n*)**	3094	224	2870
Female^c^	57.1% (1755)	43.9% (94)	58.1% (1661)
Age group^c^
18–24	21.6% (657)	6.0% (12)	22.7% (645)
25–34	37.1% (1130)	37.0% (74)	37.1% (1056)
35–44	21.9% (668)	30.5% (61)	21.3% (607)
45–54	12.7% (386)	18.5% (37)	12.3% (349)
55 and over	6.7% (204)	8.0% (16)	6.6% (188)
Body mass index (BMI)^c^
Underweight	2.1% (61)	2.6% (5)	2.1% (56)
Normal weight	49.8% (1423)	59.3% (115)	49.1% (1308)
Overweight	29.6% (845)	28.9% (56)	29.6% (789)
Obese	18.6% (531)	9.3% (18)	19.3% (513)

*^a^ Year range represents year workout was performed (for workouts and routes) or year account was created (for users). Routes were counted in whichever time period had more than 50% of the workouts using that route*.

*^b^ Other includes swimming, sports/activities, and gym/health club. There were no workouts logged in the early time period that fall into other*.

*^c^ Sex missing information on 21 users, age missing information on 49 users, and body mass index missing information on 234 users*.

Routes were used between 1 and 47 times and on average each route was utilized by only one workout. Of the 42003 unique routes, 71.3% did not enter a park at all and 2.9% were entirely within a park. On average, 11.1% (SD 27.1%) of each route was within a park. Routes by workouts from the earlier time period (2007–2009) were more likely to not enter a park at all, less likely to be entirely in a park, and had a lower percent within a park than routes by workouts occurring later (2010–2013).

Users had a mean of 14 workouts (median 5, range 1–410). Of the 3094 unique adult users, only 21, 49, and 234 were missing information on sex, age group, and BMI, respectively. A majority of users were female (57.1%), although among earlier users (joined between 2006 and 2009), men were the majority (female 43.9%). A majority of users were between the ages of 18 and 44. Just under half of the users were normal weight (49.8%), with 29.6% overweight, and 18.6% obese. Newer users had a wider age range and wider BMI range; a lower percentage of earlier users were overweight or obese and the age distribution among earlier users was slightly older.

Type of workout, distance, time taken, speed, estimated calories burned, and characteristics of the user who performed the workout (sex, age group, and BMI) varied by amount of workout route in a park (Table 2). Compared to workouts outside or partially in parks, a higher percentage of workouts entirely within parks were runs (68.8%), while a higher percent of workouts with more than half of points in parks were bicycle rides (33.5%). Overall, workouts that were partially in parks were longer, took more time, were faster in speed, and burned more estimated calories than workouts that did not enter any park or workouts that were entirely in a park. A higher percentage of workouts entirely in parks were performed by females (64.7%). The age distribution was slightly younger for workouts that were 50% or more or entirely within parks. Additionally, a higher percent of workouts that were 50% or more or entirely within parks were done by obese individuals.

**Table 2:** **Characteristics of Winston-Salem, NC, USA MapMyFitness workouts by percent of workout in parks (June 2006–September 2013)**.

	Not in park^c^	Less than half in park^c^	More than half in park^a^	Entirely in park^c^	*p*-Value^b^

	**Mean (SD) or percent (*n*) *n* = 31549**	**Mean (SD) or percent (*n*) *n* = 8127**	**Mean (SD) or percent (*n*) *n* = 2957**	**Mean (SD) or percent (*n*) *n* = 1239**	
Workout type					<0.0001
Run	62.0% (19563)	63.4% (5153)	46.6% (1378)	68.8% (852)	
Walk or hike	28.6% (9011)	22.7% (1841)	18.8% (555)	27.1% (336)	
Bicycle	7.3% (2314)	13.0% (1060)	33.5% (991)	3.5% (43)	
Other^c^	2.1% (661)	0.9% (73)	1.1% (33)	0.7% (8)	
Distance (mi)
Run, walk, or hike mean	3.1 (2.0)	3.9 (2.8)	4.2 (3.0)	3.0 (1.8)	<0.0001
Bicycle mean	12.5 (9.4)	18.3 (14.8)	12.9 (6.0)	4.8 (4.3)	<0.0001
Other mean	3.3 (3.3)	5.6 (8.4)	7.6 (6.4)	2.0 (1.6)	<0.0001
Speed (mph)
Run, walk, or hike mean	5.1 (1.7)	5.2 (1.6)	5.2 (1.7)	5.0 (1.7)	<0.0001
Bicycle mean	12.1 (4.0)	12.3 (3.6)	10.9 (3.4)	7.5 (2.7)	<0.0001
Other mean	5.1 (2.7)	6.8 (3.4)	7.1 (4.7)	4.1 (1.7)	<0.0001
Time taken (min)	43.2 (134.0)	53.1 (63.6)	62.0 (171.9)	45.7 (221.4)	<0.0001
Estimated calories burned (kcal)	353.1 (450.2)	487.8 (618.6)	589.9 (957.4)	340.6 (348.8)	<0.0001
**Characteristic of user performing workout**
Female^d^	59.5% (18702)	56.2% (4554)	49.6% (1459)	64.7% (798)	<0.0001
Age group^d^					<0.0001
18–24	14.6% (4573)	9.6% (774)	11.8% (346)	9.0% (110)	
25–34	33.9% (10594)	44.4% (3583)	29.4% (862)	30.0% (369)	
35–44	25.4% (7938)	23.7% (1914)	25.5% (748)	32.6% (401)	
45–54	15.3% (4775)	13.3% (1074)	22.7% (666)	21.9% (269)	
55 and over	10.9% (3403)	9.0% (730)	10.5% (309)	6.5% (80)	
Body mass index^d^					<0.0001
Underweight	1.8% (553)	2.8% (219)	1.9% (54)	0.9% (11)	
Normal weight	53.9% (16390)	52.2% (4086)	44.7% (1272)	50.7% (599)	
Overweight	30.9% (9397)	32.1% (2508)	32.8% (934)	27.3% (322)	
Obese	13.4% (4090)	12.9% (1012)	20.6% (586)	21.1% (249)	

*^a^ “Not in park” represents routes where no point along the route is within park boundaries. “Less than half in park” represents routes that are between >0% in parks, but <50%. “More than half in park” represents routes that are at least 50% in parks but not 100%. “Entirely in park” represents a route where 100% of points are within park boundaries*.

*^b^*p*-Value from Chi-square and ANOVA or Kruskal–Wallis non-parametric tests for categorical and continuous variables, respectively comparing across amount of workout in park*.

*^c^ Other includes swimming, sports/activities, and gym/health club*.

*^d^ Sex missing information on 141 workouts, age missing information on 354 workouts, and body mass index missing information on 1590 workouts*.

### Example summary

This example illustrates how MapMyFitness can be used to describe characteristics of physical activity episodes and for identifying parks’ influence on types of physical activity. Use of MapMyFitness grew exponentially starting in 2010. Users from the earlier time period had a narrower age and BMI range and higher average physical activity levels. Over a quarter of routes entered a park at least once during their workout (28.7%) and workout type, distance, duration, speed, and estimated calories differed across the proportion of the workout that took place in a park. Additionally, users who conducted workouts in parks were more likely to be female, were younger, and had a higher BMI than users who did not work out in parks.

This example has several limitations. By restricting to workouts in which we had corresponding routes and users, we are only examining workouts that occurred in the study area, with a linked geographic route that is also entirely within the study area, by users who indicate that they live in Winston-Salem. Therefore, this analysis does not include users who live elsewhere but may have traveled to Winston-Salem and logged a route, or who set up their account in a different location then moved to Winston-Salem and did not update their user information to Winston-Salem. We also do not know the time frequency in which GPS points were taken to create the route KML files, limiting our ability to discuss length of time a route may have spent in a park. In some instances, the park shapes (polygons) from the two data sources that we collected park information from did not exactly match. From visual inspection, and based on the names and percent of park area that matched, the same or different park was determined. This method incorporated an element of subjectivity, because we did not visit the park to visually inspect the differences.

## Advantages of this Approach

The use of MapMyFitness presents several advantages to potentially advance the field of physical activity measurement. Foremost, MapMyFitness allows for the collection of large-scale objective GPS data on the location of physical activity. GPS data have been widely recognized to be more accurate than self-reported travel surveys and activity diaries in tracking an individual’s location (28), but the high participant burden of wearing and charging GPS devices has limited the growth of these data (11). By allowing users to record GPS information through a smartphone application, MapMyFitness provides a platform to collect massive amounts of GPS data. Since data collection is passive, there is no need to ask participants to carry a separate GPS unit, which reduces burden on participants and researchers for data collection. Additionally, the MapMyFitness application is free and available on multiple devices (including iPhone, Android, Blackberry, and Windows). In the United States, where over 60% of mobile phone users own a smartphone (5, 7), this tool is available to a large number of individuals. Additionally, MapMyFitness estimates that in 2013 about 500,000 new workouts are logged around the world each day. The enormous scale of these data creates the potential to explore questions about physical activity in many more individuals, at a much more detailed level than in previous studies. MapMyFitness also alleviates concerns about low adherence, a core limitation of GPS studies (11). Researchers recognize that longer periods of study provide better information on routine physical activity, but a recent review demonstrated that data loss increases substantially after only 4 days (11). Due to low participant burden and the user desire for feedback, adherence for MapMyFitness may be on a time scale of months to years. This type of information has been elusive, and MapMyFitness may represent a breakthrough for researchers, although in Winston-Salem the amount of workouts tracked by each user varied greatly.

## Disadvantages of this Approach

Despite these major advantages, MapMyFitness does have a number of significant shortcomings for research. Primarily, the generalizability of MapMyFitness data must be thoughtfully considered before use in research. Overall, generalizability is limited by non-random sampling and missingness of: (1) who is included (i.e., using MapMyFitness), (2) which activities are included (i.e., not continual monitoring of GPS), and (3) which points are included in a route (i.e., GPS quality). Users of the application are by definition physical activity conscious, and may not be representative of the general population. Therefore, their patterns and preferences in physical activity may not be generalized to the general population. Within MapMyFitness users, there may be differences between those who use MapMyFitness regularly versus those who use it infrequently. Additionally, users may be different with regard to sociodemographics. In particular, smartphone users may be younger or have more financial resources. Using the Winston-Salem dataset above, we compared Census 2010 and (SMART BRFSS City and County) 2008 data from adult residents of the Winston-Salem Metropolitan Statistical Area (Davie County, Forsyth County, Stokes County, and Yadkin County) to MapMyFitness users’ provided information. Compared to the Census data, MapMyFitness users have a narrower age range and are more likely to be female (57.1% compared to 53.0%) (32). MapMyFitness users also had a lower prevalence of obesity (29.6% overweight and 18.6% obese compared to 39.8 and 29.1%, respectively) than identified through population-based samples (33). One further problem is the ability to make inferences on a constantly changing database. As the MapMyFitness database grows exponentially, the users, the routes, and workouts are an ever-shifting target. Thus, determining the extent to which these data are representative is challenging. This problem is compounded in research attempting to identify trends in physical activity; it is difficult to disentangle which patterns are trends in physical activity and which are trends in MapMyFitness users. Additionally, in the MapMyFitness data provided for our example, each user only had one user record. This precludes the ability to look at changes in user characteristics over time at the individual level, since we do not know whether BMI changes within the user.

Beyond the differences in MapMyFitness users compared to a population sample, discontinuous monitoring, and variations in GPS signal could create additional missingness. MapMyFitness is missing data on the location of users when they are not engaged in physical activity (e.g., not tracking a run), and more specifically, when they are not engaged in the physical activities tracked by MapMyFitness or are engaged in physical activity but choose not to track it using MapMyFitness. Therefore, GPS data from the application do not provide a complete picture of overall daily physical activity. In practice, researchers could ask participants to leave MapMyFitness on the entire day. However, due to battery constraints of typical smartphones, MapMyFitness is not intended for use throughout the day. Other apps, including Moves^2^, may accomplish this research aim. As with any other GPS device, signal dropout is a concern with MapMyFitness, and the quality of these data may vary, especially in urban areas where GPS signal acquisition can suffer (11). Additionally, GPS accuracy from smartphones may be different than GPS accuracy from a devoted GPS logger. Finally, since users can log routes in multiple ways there are potential measurement differences by GPS device (e.g., Garmin watch compared to smartphone GPS). Additionally, when routes are logged online, this creates a route KML file similar to one logged using GPS. However, a user may not follow the exact path they planned online. The dataset used in our example did not have an indicator as to whether routes were tracked by GPS device, GPS within the app, or manually within the website interface. Teasing apart which routes are logged via GPS and which were logged online through the website would be critical to knowing the accuracy of the mapped route. Furthermore, GPS data may lack some of the objective, contextually rich information that can be gained through direct observation tools, such as SOPARC (34).

The validity of user-input data is also a concern. BMI is based on self-report, which has known issues with misclassification (35). BMI is also entered by the user upon the initial installation of the application; however, it is unlikely that this information is ever updated. Additionally, it is unclear whether age is updated over time or whether the user’s baseline age at first download is constant in the dataset.

One of the largest drawbacks of using MapMyFitness for research is the dual role of MapMyFitness as both a tracking technology and a potential behavior-altering intervention. People may choose to run farther or along different routes while they are using MapMyFitness than if they were running without the technology. MapMyFitness’ “MVP” users have access to workout plans and suggested routes in order to assist in reaching their fitness goals. Additionally, MapMyFitness encourages users to be more physically active through competitions. We were not provided with the proportion of Winston-Salem users who are MVP or an indicator of which routes may have been logged as part of competitions, so this could not be accounted for in our analyses.

The current MapMyFitness global route database is multiple terabytes (1 TB = 1000 GB) and grows each day, making processing challenging. Even given the limited geographic scope of Winston-Salem, NC, USA we had several computing issues due to the large size of MapMyFitness data. Processing times for combining route information with park information took upwards of 3 weeks using a desktop Windows operating system. Researchers attempting to utilize these types of data may be best suited with an interdisciplinary team that includes contribution from experienced geographers, computer scientists, and biostatisticians.

## Ethical Considerations

As a new avenue of research, utilizing health data from citizens tracking it for personal purposes brings up numerous ethical questions. The Health Data Exploration project is examining these unique scientific, methodological, and ethical issues with support from The Robert Wood Johnson Foundation (36). As of October 2013, when we obtained MapMyFitness data, this project was surveying and interviewing individuals, researchers, and companies in order to understand and convey some of the best practices for handling this type of data. In the absence of guidelines for best practice, we proceeded with caution around the use of this data.

The example in this report was approved and deemed exempt by the University of Michigan Institutional Review Board. However, ethical considerations are a fundamental concern when working with open access GPS data available through MapMyFitness. Although identifying information is not provided, such as user names and addresses, GPS data have the potential to reveal timing patterns of visits to certain locations and even home locations. MapMyFitness is working to ensure that users are protected when researchers access the location of their workouts, and other potentially sensitive personal information, such as BMI and age. It is important to note that MapMyFitness does not currently provide individual data to commercial interests. Therefore, care will need to be taken to confirm that data use is solely within the research domain. Researchers who use MapMyFitness data should take caution to aggregate results before they are presented, published, or shared. Special attention should be paid to identifiability when creating maps of workouts for a given area.

## Perspectives

MapMyFitness is a powerful tool to investigate patterns of physical activity in a broader population across a large geographic and temporal scope. As self-tracking becomes increasingly prevalent across the United States and the world, incorporation of these types of technologies will allow researchers to explore more complex and comprehensive questions. Additional work is needed to understand best practices for data sharing, security, storage, and processing. The large data size precipitates the need for new methods that will only be successful through collaboration with researchers in engineering or computer science. Clarifying the roles of private companies in research and exploring the ethics around user data will be critical for advancement of the use of technology in physical activity research.

## Conflict of Interest Statement

Kyler Eastman works for MapMyFitness, Inc., the company who produces the MapMyFitness suite of apps. His role on the paper was to advise on the data structure, assist with clarifying MapMyFitness data questions, subset the Winston-Salem dataset from the MapMyFitness data, confirm that material in the methods article is accurate, and protect the privacy of MapMyFitness users. The remaining authors declare no conflicts of interest.

## Footnotes

^1^http://about.mapmyfitness.com/

^2^http://www.moves-app.com
